# Behavioral changes in patients with Prader-Willi syndrome receiving diazoxide choline extended-release tablets compared to the PATH for PWS natural history study

**DOI:** 10.1186/s11689-024-09536-x

**Published:** 2024-04-26

**Authors:** Theresa V. Strong, Jennifer L. Miller, Shawn E. McCandless, Evelien Gevers, Jack A. Yanovski, Lisa Matesevac, Jessica Bohonowych, Shaila Ballal, Kristen Yen, Patricia Hirano, Neil M. Cowen, Anish Bhatnagar

**Affiliations:** 1 Foundation for Prader-Willi Research, Covina, CA 91723 USA; 2 University of Florida College of Medicine, Gainesville, FL 32610 USA; 3 University of Colorado School of Medicine and Children’s Hospital Colorado, Aurora, CO 80045 USA; 4 Queen Mary University of London and Barts Health NHS Trust – Royal London Children’s Hospital, London, E1 1FR UK; 5 US Eunice Kennedy Shriver National Institute of Child Health and Human Development, National Institute of Health, Bethesda, MD 20847 USA; 6 Soleno Therapeutics, Redwood City, CA 94065 USA

**Keywords:** Prader-Willi syndrome, Hyperphagia, Natural history, DCCR

## Abstract

**Background:**

Prader-Willi syndrome (PWS) is a rare neurobehavioral-metabolic disease caused by the lack of paternally expressed genes in the chromosome 15q11-q13 region, characterized by hypotonia, neurocognitive problems, behavioral difficulties, endocrinopathies, and hyperphagia resulting in severe obesity if energy intake is not controlled. Diazoxide choline extended-release (DCCR) tablets have previously been evaluated for their effects on hyperphagia and other behavioral complications of people with PWS in a Phase 3 placebo-controlled study of participants with PWS, age 4 and older with hyperphagia (C601) and in an open label extension study, C602.

**Methods:**

To better understand the longer-term impact of DCCR, a cohort from PATH for PWS, a natural history study that enrolled participants with PWS age 5 and older, who met the C601 age, weight and baseline hyperphagia inclusion criteria and had 2 hyperphagia assessments ≥ 6 months apart, were compared to the C601/C602 cohort. Hyperphagia was measured using the Hyperphagia Questionnaire for Clinical Trials (HQ-CT, range 0–36). The primary analysis used observed values with no explicit imputation of missing data. A sensitivity analysis was conducted in which all missing HQ-CT assessments in the C601/C602 cohort were assigned the highest possible value (36), representing the worst-case scenario. Other behavioral changes were assessed using the Prader-Willi Syndrome Profile questionnaire (PWSP).

**Results:**

Relative to the PATH for PWS natural history study cohort, the DCCR-treated C601/C602 cohort showed significant improvements in HQ-CT score at 26 weeks (LSmean [SE] -8.3 [0.75] vs. -2.5 [0.43], *p* < 0.001) and 52 weeks (LSmean [SE] -9.2 [0.77] vs. -3.4 [0.47], *p* < 0.001). The comparison between the cohorts remained significant in the worst-case imputation sensitivity analysis. There were also significant improvements in all domains of the PWSP at 26 weeks (all *p* < 0.001) and 52 weeks (all *p* ≤ 0.003) for C601/C602 participants compared to the PATH for PWS participants.

**Conclusion:**

Long-term administration of DCCR to people with PWS resulted in changes in hyperphagia and other behavioral complications of PWS that are distinct from the natural history of the syndrome as exemplified by the cohort from PATH for PWS. The combined effects of administration of DCCR should reduce the burden of the syndrome on the patient, caregivers and their families, and thereby may benefit people with PWS and their families.

**Trial Registration:**

Clinical study C601 was originally registered on ClinicalTrials.gov on February 22, 2018 (NCT03440814). Clinical study C602 was originally registered on ClinicalTrials.gov on October 22, 2018 (NCT03714373). PATH for PWS was originally registered on ClinicalTrials.gov on October 24, 2018 (NCT03718416).

## Background

Prader–Willi syndrome (PWS) is a rare, complex genetic neurobehavioral/metabolic disorder with an estimated birth incidence of 1:15,000 to 1:20,000 [1, 2]. PWS arises from lack of expression of paternally inherited imprinted genes on chromosome 15q11-q13 caused by a paternal deletion, maternal uniparental disomy 15 or an imprinting center defect, resulting in hypothalamic dysfunction [3]. Clinical features of PWS include hypotonia and feeding difficulties in infancy and sustained accumulation of excess body fat beginning in early childhood [4]. Hyperphagia, which occurs at a median age of 8 years, presents as food obsession, aggressive food seeking, and lack of satiety, with progression to severe obesity if energy intake is not restricted [4]. PWS is also associated with intellectual disability, low muscle mass, neuroendocrine abnormalities including growth hormone and gonadotropin deficiency, behavioral problems including aggression, anxiety and compulsivity, and elevated risk for early mortality [5–7]. According to surveys of caregivers of individuals with PWS, reducing hunger and improving food-related behaviors were the most important unmet needs in PWS that should be addressed in the development of a new therapeutic [8, 9]. There are no effective treatments for hyperphagia or other behavioral problems in PWS, although several other investigational products have been evaluated in clinical studies [10].

Diazoxide is a potent activator of the ATP-sensitive potassium (K_ATP_) channel and is capable of crossing the blood-brain barrier [11]. Activating the K_ATP_ channel in neuropeptide Y (NPY)/Agouti Related-Protein (AgRP) neurons in the hypothalamus results in reduced secretion of NPY and AgRP, potent endogenous appetite stimulatory neuropeptides, potentially contributing to a reduction in hyperphagia [12]. These actions of the drug are complemented by activating the K_ATP_ channel in the dorsal motor nucleus of the vagus nerve (amplification of the effects of insulin, leptin and alpha-melanocyte stimulating hormone on pancreatic function, insulin resistance and satiety), pancreatic β-cells (reductions in hyperinsulinemia), and adipocytes (reduction in fatty acid biosynthesis and increased beta-oxidation of fat) to reduce hyperinsulinemia and accumulation of excess body fat, and to improve insulin and leptin resistance, and satiety [12]. These effects have been confirmed in multiple animal models of genetic or induced hyperphagic obesity including a model of PWS (Magel-2 null mouse) [12].

Diazoxide choline extended release tablets (DCCR) is an extended-release tablet formulation of a highly soluble salt of diazoxide which upon administration results in release and absorption of active drug throughout the gastrointestinal tract over 24 h, contributing to very stable intraday circulating drug levels with once-per-day dosing.

This study involves a comparison of a long-term interventional trial of DCCR in PWS (C601/C602) [13, 14] to a natural history study. Natural history studies in rare diseases serve multiple purposes. First and foremost, they inform our understanding of the progression, complexity and burden of the rare disease. Secondly, provided there is comparability of enrolled subjects, and the same or similar endpoints are measured in the natural history study as are measured in interventional trials in the disease, then the natural history study may function as an appropriate and informative control cohort to the interventional study. In this role the natural history study has several advantages. Natural history studies tend to be larger than control arms in randomized studies, such that comparisons to the natural history study can be made with more power than might be achieved in the comparison to a placebo control arm. Natural history studies tend to be run longer than it may be feasible or ethical to run a placebo control arm in a randomized study, allowing for evaluation of efficacy over longer durations of administration of experimental drugs. Prospectively specifying a natural history study as the control to an interventional study likely allows the interventional study to be smaller since it does not include a placebo control arm. Consequently, the interventional study may recruit more quickly and be completed at lower cost. Finally, a single natural history study may serve as an appropriate control arm to multiple interventional studies.

DESTINY PWS (C601) was a 13-week double-blind, placebo-controlled study in people with PWS age 4 and older comparing DCCR administration to placebo [13]. In C601, using data obtained prior to the onset of the COVID19 pandemic, 13 weeks of treatment with DCCR resulted in significant improvements in hyperphagia as measured by the Hyperphagia Questionnaire for Clinical Trials (HQ-CT), in Clinical Global Impression of Improvement (CGI-I), in body fat, and in a range of other behavioral endpoints as measured by domains of the Prader-Willi Syndrome Profile (PWSP) Questionnaire [13]. In the full dataset including data collected during the pandemic, the caregiver assessed endpoints including HQ-CT did not remain significant [13]. C602 is an open label extension of the C601 study, which opened to participants upon completion of C601 [14].

In this report, we compare caregiver-assessed behavioral outcomes in patients with PWS who were given DCCR in clinical trials (C601/C602) to findings of people with PWS who participated in an independent natural history study, the “Paving the way for Advances in Treatments and Health for PWS” (PATH for PWS) study. We hypothesized that hyperphagia and other aspects of behavior often observed in PWS would show improvements among those who were administered DCCR compared to those enrolled in the natural history study.

## Methods

Participants from two independent research cohorts were compared. DESTINY-PWS (C601; NCT03440814) was an international, randomized, double-blind, placebo-controlled, parallel-group, Phase 3 study comparing DCCR to placebo in individuals with PWS age 4 years and older with hyperphagia. Dosing in this study was weight-based and targeted an average of 4.2 mg/kg [13]. Following the completion of the double-blind study, participants who sought continued treatment with DCCR were eligible to enroll in clinical study C602 (NCT03714373), a long-term open-label treatment study [14]. PATH for PWS (NCT03718416) is an ongoing natural history study, which enrolled individuals with PWS 5 years and older and was conducted by the Foundation for Prader-Willi Research (FPWR). The objectives of PATH for PWS include advancing the understanding of serious medical events in PWS over a 4-year period, as well as to evaluate how PWS related behaviors change over time. The data from this study are intended to inform the development of, and clinical trial designed for, potential new treatments for PWS. Prior to initiating each study (C601, C602 and PATH for PWS), ethics committee review and approval of the protocol and study related documents was completed. Protocols and study related materials for clinical studies C601 and C602 were reviewed and approved by the WCG Institutional Review Board. The protocol and study related materials for PATH for PWS were reviewed and approved by Hummingbird Institutional Review Board, and the study was subsequently overseen by the North Star Review Board. Participants or their parents/guardians provided informed consent and, as appropriate, assent, prior to being enrolled in each study.

Participants in PATH for PWS were recruited concurrently with the recruitment of participants in C601, providing a contemporaneous population of individuals with PWS from which a cohort could be selected that is comparable to participants randomized in C601, to compare changes in behavior over time.

A statistical Clinical Research Organization (CRO) independent of both Soleno and FPWR prepared and obtained approval from the sponsor for a statistical analysis plan (SAP) prior to the conduct of any analyses. The CRO utilized an unblinded biostatistician who identified participants for inclusion in the PATH for PWS cohort. Once the cohorts were identified and the SAP was approved the CRO was responsible for the analysis of the data. Data from clinical study C601 had already been unblinded, as had a portion of the data from the first 13 weeks of C602, but Soleno was blinded to most of the data from C602 prior to the start of statistical analysis for this comparison. A cohort of participants was identified by the statistical CRO from the PATH for PWS study, which met the key inclusion criteria for C601: baseline HQ-CT score ≥ 13, age 4 years and older, weight between 20 and 135 kg, not enrolled in an interventional clinical study at the first of two consecutive visits approximately 6 months apart with HQ-CT data available at both visits. Most of this cohort also had HQ-CT and data for other assessments available at a subsequent visit, which occurred approximately 1 year after the first visit.

The HQ-CT is a 9-item (range 0–36), caregiver completed questionnaire intended to assess a range of hyperphagia-related behaviors in PWS over a 2-week recall period including: food seeking behaviors, tendency toward being upset or distressed when denied food or asked to stop talking about food and the extent to which these behaviors interfere with daily life [15, 16]. The HQ-CT has been developed and validated for use in the assessment of hyperphagia related behaviors in clinical studies in PWS [13, 17]. The PWSP is a caregiver completed questionnaire that assesses PWS-related behaviors including 51 items which comprise 6 behavioral domains: aggressive behaviors (9 items, range 0–18), anxiety (11 items, range 0–22), compulsivity (10 items, range 0–20), depression (5 items, range 0–10), disordered thinking (6 items, range 0–12) and rigidity/irritability (10 items, range 0–20) [18]. It is intended for use in clinical trials.

Baseline for participants in the C601/C602 cohort was defined as the last measurement of the parameter made prior to initiating DCCR administration. For participants in the DCCR arm of C601, baseline is the C601 baseline measurement, while for participants in the C601 placebo arm, baseline is the end-of-treatment measurement from C601. Hereafter, weeks refer to weeks of DCCR administration. The primary endpoint was HQ-CT change from baseline at 26 weeks. Additional endpoints included HQ-CT change from baseline at 52 weeks, HQ-CT change from baseline at 26 weeks by subgroup of age (< 18 years, ≥ 18 years), PWS genetic subtype (deletion, non-deletion), sex (female, male), growth hormone treatment status (use at both visits, no growth hormone use), and baseline HQ-CT total score (≥ 18, < 18), and changes in behavioral complications of PWS assessed using the PWSP at 26 and 52 weeks.

All statistical tests were performed with a two-sided significance level of α = 0.05. Analyses were based on observed data without imputation for missing results. Comparability of the cohorts was assessed with respect to baseline and demographic characteristics. The primary comparison was the least square mean change from baseline in HQ-CT Total Score at Week 26 between the C601/C602 and the PATH for PWS cohorts from an analysis of covariance model adjusting for baseline HQ-CT Total Score and cohort. Subgroup analyses of HQ-CT change from Baseline were based on an analysis of covariance model adjusting for baseline HQ-CT Total Score and cohort. In addition, all available HQ-CT scores through Week 52 were analyzed using a mixed model for repeated measures with unstructured covariance, baseline HQ-CT, and post-baseline timepoint with an interaction term between cohort and post-baseline timepoint. A sensitivity analysis was conducted in which each missing HQ-CT assessment in the C601/C602 cohort was imputed with the highest possible score for the HQ-CT, including individuals missing data at both 26 and 52 weeks, and would not have been included in the analysis of the primary endpoint (an HQ-CT score of 36, i.e., the worst-case scenario). The resulting dataset was analyzed using the same mixed model for repeated measures as was used for the observed cases. PWSP domains were compared between cohorts based on the Mann-Whitney test. The domains of the PWSP at Week 26 and at Week 52 were also analyzed similarly to the primary ANCOVA for the HQ-CT total Score at Week 26, using their respective baseline scores as covariates.

The lack of randomization when comparing with observational cohorts may result in large differences or imbalances in the values of their respective baseline covariates that can create biased estimates of treatment effects. A methodology used to overcome such biases is the use of propensity scores. Several covariates that have been thought to affect the efficacy outcome as based on scientific rationale or known to influence efficacy as demonstrated in previous studies are considered for inclusion in propensity scores. These propensity scores are then incorporated in the statistical analysis in lieu of multiple covariates. Propensity scores combine all the information from the covariates into a single number that represents the probability of being assigned to the treatment given the covariates. A subset of subjects with similar propensity scores will tend to be similar with respect to values of the baseline and demographic characteristic.

Propensity score adjusted ANCOVA analyses were also conducted. Propensity scores were derived from a logistic regression model adjusting for age, gender, baseline weight (kg), baseline HQ-CT, growth hormone treatment status (on treatment vs. not), region (US vs. Outside the US [OUS]), and PWS genetic type (deletion vs. non-deletion).

Given the concurrent recruitment in the same or similar geographic regions and the use of the same age, weight and HQ-CT inclusion criteria, there was an expectation that the individuals in the two cohorts would be receiving a similar standard of care, and have similar durations of disease and disease severity, limiting the potential for introduction of a bias from these sources in the analysis.

## Results

**Fig. 1:**
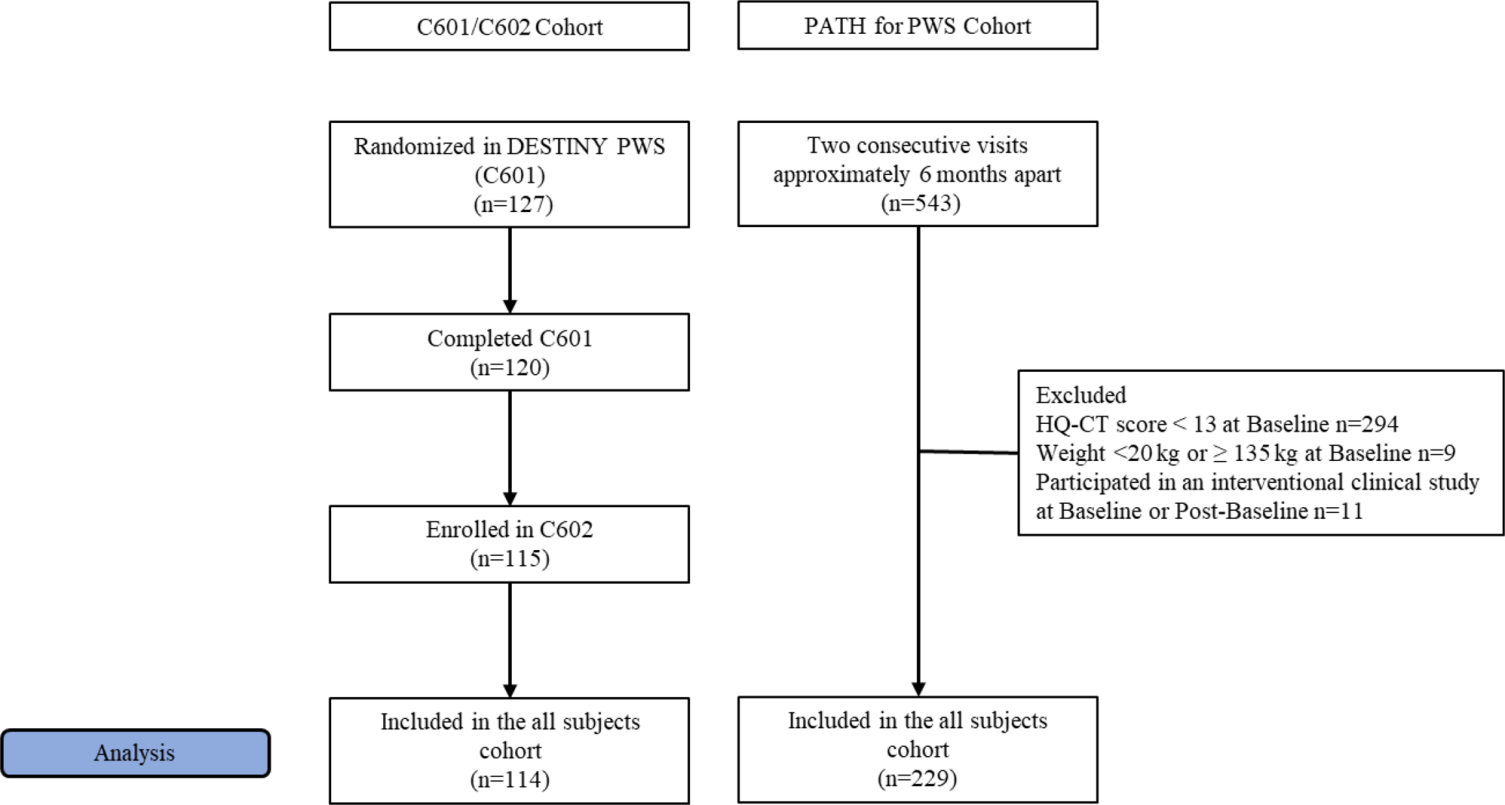
CONSORT diagram for C601/C602 and PATH for PWS cohorts

From May 2018 through January 2020, 127 participants were randomized in C601 (Fig. 1). One hundred twenty participants completed C601 and 115 enrolled in C602. Data at 26 weeks was available for 114 participants. Recruitment for PATH for PWS was initiated in October 2018 and had reached the target of about 700 participants by the time recruitment was completed for C601. Five hundred forty-three participants in PATH for PWS had data on HQ-CT from two consecutive visits approximately 6 months apart. Of these, 303 participants did not meet the inclusion criteria for age, baseline HQ-CT score or weight, and a further 11 participants were concurrently enrolled in an interventional clinical study at baseline or post-baseline (Fig. 1). Consequently, the cohort from PATH for PWS consisted of 229 participants. Of these 229 participants in PATH for PWS, 197 also had data available at approximately 52 weeks. Demographic and baseline characteristics for the two cohorts are presented in Table 1. The two cohorts are comparable, with the primary differences between the cohorts being that the PATH for PWS cohort was slightly older, and, consequently, had somewhat lower rates of growth hormone treatment, and incrementally lower baseline HQ-CT scores. Thus, the PATH for PWS cohort was deemed to effectively serve as an external natural history control for the cohort from C601/C602.

**Table 1:** Demographics and Baseline Characteristics of Participants in C601/C602 and PATH for PWS

	C601/C602 (*N* = 114)	PATH for PWS (*N* = 229)
**Age, years mean ± SD**	13.1 ± 6.17	17.9 ± 9.49
**Race, n (%)**		
White	98 (86.0%)	185 (80.8%)
Black or African American	6 (5.3%)	6 (2.6%)
Asian	1 (0.9%)	4 (1.7%)
American Indian or Alaska Native	0 (0.0%)	0 (0.0%)
Native Hawaiian or Other Pacific Islander	0 (0.0%)	0 (0.0%)
Other	2 (1.8%)	9 (3.9%)
Multiple	7 (6.1%)	22 (9.6%)
Not Reported	0 (0.0%)	3 (1.3%)
**Sex, n (%)**		
Male	48 (42.1%)	110 (48.0%)
Female	66 (57.9%)	119 (52.0%)
**PWS genetic subtype n (%)**		
Deletion	71 (62.3%)	113 (49.3%)
Non-deletion	43 (37.7%)	82 (35.8%)
Not reported	0 (0.0%)	34 (14.8%)
**Weight, kg mean ± SD**	60.3 ± 29.69	66.7 ± 26.72
**BMI, mean ± SD**	26.8 ± 9.3	28.9 ± 9.76
**Baseline HQ-CT, mean ± SD**	21.3 ± 6.73	18.2 ± 4.99
**Growth hormone use n (%)**		
Yes (at both visits)	95 (83.3%)	130 (56.8%)
No (at both visits)	19 (16.7%)	87 (38%)
Only at baseline	0 (0.0%)	6 (2.6%)
Only at 6 months	0 (0.0%)	6 (2.6%)
**PWSP Domain**	*N* = 103	*N* = 167
Aggressive Behaviors mean ± SD	7.5 ± 3.81	8.9 ± 4.18
Anxiety mean ± SD	12.9 ± 5.29	13.7 ± 5.05
Compulsivity mean ± SD	12.0 ± 4.85	12.3 ± 4.48
Depression mean ± SD	3.6 ± 2.27	3.8 ± 2.25
Disorder Thinking mean ± SD	4.3 ± 3.33	4.5 ± 3.05
Rigidity/Irritability mean ± SD	10.9 ± 5.33	12.0 ± 5.25

### HQ-CT

**Fig. 2:**
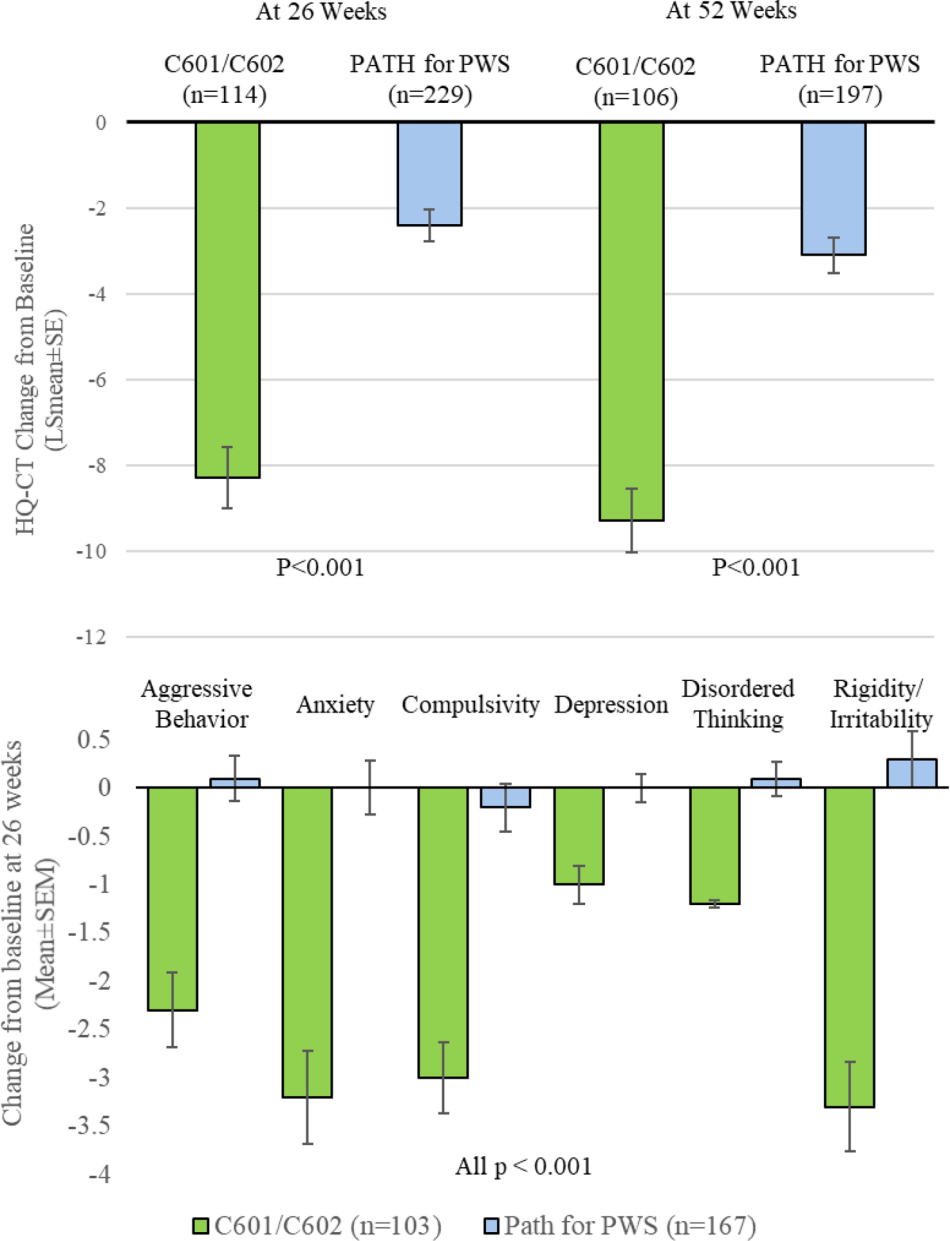
**A** HQ-CT Change from Baseline Comparison of C601/C602 to PATH for PWS at 26 Weeks and 52 Weeks; and 2**B**. Prader-Willi Syndrome Profile Domain Change from Baseline Comparison of C601/C602 to PATH for PWS at 26 Weeks

DCCR administration in C601/C602 resulted in a statistically significantly greater HQ-CT change from baseline compared to PATH for PWS at both 26 weeks (Fig. 2A, LS mean [SE] -8.3 [0.71] vs. -2.4 [0.43], *p* < 0.001) and 52 weeks (Fig. 2A, LS mean [SE] -9.3 [0.77] vs. -3.1 [0.42], *p* < 0.001). There was a significantly greater HQ-CT change from baseline at 26 weeks in the C601/C602 cohort compared to the PATH for PWS cohort in all planned subgroups of age, sex, PWS subtype, growth hormone use, region, and baseline HQ-CT score (Table 2). In the propensity quintile adjusted ANCOVA analysis, DCCR in C601/C602 resulted in a statistically significantly greater HQ-CT change from baseline compared to PATH for PWS at 26 weeks (Table 3, *p* < 0.001) and the worst-case sensitivity analysis was also significant at 26 and 52 weeks (Table 3).

**Table 2:** HQ-CT Change from Baseline at Week 26 Subgroup Analyses Comparing C601/C602 Cohort to PATH for PWS Cohort

	Number of participants		LS Mean (95% CI)		LS Mean Difference (95% CI)*	P-value
Subgroup	C601/ C602	PATH	C601/C602	PATH		
**Age**						
< 18	91	129	-8.7 (-10.1, -7.3)	-3.0 (-4.2, -1.8)	-5.7 (-7.6, -3.8)	< 0.001
≥ 18	17	100	-7.4 (-10.1, -4.7)	-1.5 (-2.6, -0.3)	-6.0 (-8.9, -3.0)	< 0.001
**Sex**						
Female	63	119	-8.2 (-9.8, -6.6)	-2.9 (-4.1, -1.8)	-5.3 (-7.4, -3.3)	< 0.001
Male	45	110	-8.5 (-10.4, -6.5)	-1.8 (-3.1, -0.6)	-6.6 (-9.0, -4.3)	< 0.001
**PWS Subtype**						
Deletion	69	113	-9.2 (-10.8, -7.7)	-2.1 (-3.3, -0.9)	-7.1 (-9.1, -5.1)	< 0.001
Non-Deletion	39	82	-7.2 (-9.4, -5.0)	-2.8 (-4.3, -1.4)	-4.3 (-7.1, -1.6)	0.002
**Growth Hormone Use**						
Both Visits	93	130	-8.6 (-10.1, -7.2)	-2.8 (-4.0, -1.6)	-5.8 (-7.7, -3.9)	< 0.001
Untreated	15	87	-6.8 (-9.8, -3.8)	-1.9 (-3.1, -0.6)	-4.9 (-8.2, -1.7)	0.004
**Region**						
United States	86	202	-9.2 (-10.6, -7.8)	-2.6 (-3.4, -1.7)	-6.6 (-8.3. -5.0)	< 0.001
Ex-United States	22	27	-5.4 (-8.3, -2.4)	-0.8 (-3.5, 1.8)	-4.5 (-8.6, -0.5)	0.028
**Baseline HQ-CT**						
Above Median (≥ 18)	75	103	-10.7 (-12.3, -9.1)	-2.6 (-3.9, -1.2)	-8.1 (-10.3, -6.0)	< 0.001
Below Median (< 18)	33	126	-4.4 (-6.3, -2.5)	-1.8 (-2.8, -0.9)	-2.6 (-4.7, -0.4)	0.019

*Difference in LS Means and 95% confidence intervals are derived from an ANCOVA model with baseline value as a covariate

In C601, a minimally clinically meaningful change in HQ-CT was defined as a reduction in HQ-CT score of 7 points [13]. At 26 weeks, there was a statistically significant difference in percentage of participants experiencing at least a 7-point reduction HQ-CT in C601/C602 compared to PATH FOR PWS (60% vs. 20.5%, OR [95% CI] 0.20 [0.12, 0.36], *p* < 0.001).

### Behavioral endpoints

There were broad-ranging improvements in non-food-related behavioral complications of PWS with statistically significant results obtained for all domains of the PWSP including aggressive behaviors, anxiety, compulsivity, depression, disordered thinking and rigidity/irritability in the C601/C602 cohort compared to the PATH for PWS cohort at 26 weeks (Fig. 2B, all *p* < 0.001). Similar results were observed at 52 weeks (Table 3, all domains *p* < 0.001 except depression domain *p* = 0.003) (Table 3).

**Table 3:** Comparison of C601/C602 to PATH for PWS Change from Baseline at 26, and 52 Weeks

Parameter		C601/C602		PATH for PWS		
HQ-CT	Measured at	n	LSmean [SE]	n	LSmean [SE]	p-value
Propensity quintiles	26 weeks	108	-8.3 [0.75]	195	-2.5 [0.43]	< 0.001
Worst-Case Sensitivity analysis	26 weeks	123	-5.4 [0.97]	229	-2.5 [0.39]	0.006
Worst-Case Sensitivity analysis	52 weeks	123	-5.9 [1.04]	197	-3.1 [0.43]	0.015
**PWSP domain**	**Measured at**	**n**	**LSmean [SE]**	**n**	**LSmean [SE]**	**p-value**
Aggressive Behavior	52 weeks	103	-2.1 [0.31]	147	0.3 [0.26]	< 0.001
Anxiety	52 weeks	103	-3.2 [0.35]	147	0.1 [0.30]	< 0.001
Compulsivity	52 weeks	103	-3.0 [0.30]	147	-0.2 [0.25]	< 0.001
Depression	52 weeks	103	-1.0 [0.18]	147	-0.1 [0.15]	0.003
Disordered Thinking	52 weeks	103	-1.4 [0.20]	147	0.01 [0.17]	< 0.001
Rigidity/ Irritability	52 weeks	103	-3.1 [0.36]	147	-0.2 [0.30]	< 0.001

## Discussion

Compared to the natural history of PWS as exemplified by the PATH for PWS cohort, the DCCR cohort had improved hyperphagia, the hallmark of PWS, measured by the HQ-CT at both 26 weeks and 52 weeks in the whole population and in all subgroup analyses at 26 weeks. Similar magnitudes of improvement in HQ-CT in the C601/C602 cohort compared to the PATH for PWS cohort were realized in both pediatric and adult participants, in females and males, and in growth hormone treated and naïve subgroups. Consistent with the results of C601 and in long-term treatment with DCCR in C601/C602, greater improvements relative to the natural history of PWS were realized with DCCR in those with more severe hyperphagia at baseline [13]. The observed improvements in hyperphagia relative to the natural history of PWS likely resulted from the combination of the direct effects of DCCR in NPY/AgRP neurons and from improvements in insulin and leptin resistance [11]. The magnitude of improvement in HQ-CT at 26 weeks in this analysis was greater than that observed at 13 weeks in C601 [13]. The comparison between cohorts remains significant, even when all missing values including those for subjects in the C601/C602 cohort that were missing both the 26 week and 52 week measurements, were assigned the highest possible score for HQ-CT (the worst case scenario), confirming the robustness of the results.

Strengths of this study include the contemporaneous accrual of participants who completed the same measures in the DCCR C601/C602 studies and the natural history PATH for PWS study. Limitations are primarily related to the fact that there was open-label administration of DCCR in C602, potentially allowing improvements to be ascribed to the drug when they were primarily due to being in an interventional, rather than an observational, study, and the secondary nature of this analysis of two independent cohorts.

DCCR administration in C601/C602 resulted in broad-ranging impacts on PWS-associated behaviors as measured using the PWSP, with the comparison of change from baseline in DCCR participants in C601/C602 to the results from PATH for PWS being significant for all domains at both 26 and 52 weeks. Improvements in anxiety, compulsivity, aggressive behaviors, rigidity/irritability, disordered thinking and depression were observed with DCCR administration relative to the natural history of PWS. These results suggest that DCCR administration to participants with PWS improves the most important behavioral complications of the condition relative to the natural history of the disease.

## Conclusions

DCCR administration resulted in changes in hyperphagia and other behavioral complications of PWS that are significantly improved from the natural history of the syndrome as exemplified by the cohort from PATH for PWS. The combined effects of long-term administration of DCCR should reduce the burden of the syndrome on the patient, caregivers and their families, and thereby may benefit people with PWS and their families.

## Data Availability

The C601/C602 the data sets generated and/or analyzed during the current study are not publicly available but are available from Soleno Therapeutics for further research on reasonable request. Data from the PATH for PWS are not publicly available but are available from FPWR for further research on reasonable request.
